# Surface modification of gold nanoparticles with 6-mercapto-1-hexanol to facilitate dual conjugation of protoporphyrin IX and folic acid for improving the targeted photochemical internalization 

**DOI:** 10.22038/IJBMS.2022.63622.14033

**Published:** 2022-08

**Authors:** Armin Imanparast, Neda Attaran, Hossein Eshghi, Ameneh Sazgarnia

**Affiliations:** 1 Medical Physics Research Center, Mashhad University of Medical Sciences, Mashhad, Iran; 2 Department of Medical Nanotechnology, Applied Biophotonics Research Center, Tehran Science and Research Branch, Islamic Azad University, Tehran, Iran; 3 Department of Chemistry, Faculty of Science, Ferdowsi University of Mashhad, Mashhad, Iran

**Keywords:** 6-mercapto-1-hexanol, Cervical cancer, Folic acid, Gold nanoparticle, Photochemical – internalization, Photodynamic therapy, Protoporphyrin-IX

## Abstract

**Objective(s):**

Photochemical internalization (PCI) is an important type of photodynamic therapy for delivering macromolecules into the cytosol by the endocytosis process. In this study, 6-mercapto-1-hexanol (MH) was used to functionalize the gold nanostructure as a primer for surface modification to improve conjugation of multi-agents such as protoporphyrin IX (Pp-IX) and folic acid with gold nanoparticles (PpIX/FA-MH-AuNP) to facilitate the photochemical internalization.

**Materials and Methods:**

After surface modification of AuNPs with MH, PpIX and FA are bonded to the surface of the MH-AuNPs through the coupling reaction to produce the desired conjugated AuNPs. In the next step, the synthesized nanostructures were characterized by different methods. Finally, after selecting specific concentrations, light treatments were applied and cell survival was measured based on MTT analysis. Also, in order to better study the morphology of the cells, they were stained by the Giemsa method. The SPSS 16 software was used for data analysis

**Results:**

By surface modification of the nanostructure with MH and then conjugation of FA to it, the incubation time of the drug in PpIX/FA-MH-AuNP was reduced from 3 hr to 30 min. Also, at each light dose, cell death in the presence of PpIX/FA-MH-AuNP was significantly reduced compared with unconjugated conditions (*P*<0.001). Under these conditions, the ED50 for PpIX and PpIX-MH-AuNP and PpIX/FA-MH-AuNP at a concentration of 2.5 μg/ml is 8.9, 9.1, and 6.17 min, respectively.

**Conclusion:**

The results show that the PCI of PpIX/FA-MH-AuNP increases the selective phototoxicity efficiency on cancer cells compared with the conventional process of photodynamic therapy.

## Introduction

Photodynamic therapy (PDT) is one of the treatment methods based on non-ionizing radiation which is non-invasive and can selectively remove cancerous cells. In order to initiate the process of photodynamic therapy, three essentials are required, which include the photosensitizer (PS), microenvironment molecular oxygen level, and overlap of the absorption peak of PS with an emission spectrum of a light source (1). These factors are nontoxic individually, but the combined use leads to the induction of photochemical cytotoxicity (2). The products of the photodynamic process include species derived from free radicals, of which singlet oxygen (^1^O_2_) and reactive oxygen species (ROS) are two important components. The increase of free radicals in cancer cells leads to cell death through apoptosis or necrosis (3).

The therapeutic efficiency of current photodynamic treatment methods is limited due to several reasons (4). Two important challenges include non-specific targeting of desired cells and lack of effective cell uptake due to photosensitizer entrapment inside the endolysosomal compartment (4-6).

Once the photosensitizer reaches the target cell, it must undergo an internalization process to reach its primary site of action inside the cell. In this regard, the plasma membrane is a lipid barrier that manages the selective entry of chemical/photosensitizing agents into the cell (7). The photosensitizers or drug nanocarriers are normally internalized by endocytosis, which results in their being placed inside an endolysosomal compartment, thus limiting their interaction with the target molecule (8, 9). 

One of the effective methods to overcome endolysosomal entrapment are the use of physicochemical enhancing techniques. In these techniques, disrupting the membrane and releasing the contents of the endolysosomal compartment causes physical changes or induction of chemical reactions. Some of these methods include the use of magnetic fields (10), ultrasounds (11), and photochemical internalization (12). 

Photochemical internalization (PCI) is an important type of PDT for delivering macromolecules into the cytosol by the endocytosis process. In this technique, by stimulating the photosensitizer with light photons and producing free radicals, endolysosomal membranes are disrupted and its contents (including nanoparticles carrying the photosensitizer) are released. PCI has been shown to facilitate the biological activity and selective toxicity of many macromolecules that are difficult to pass through the plasma membrane (13, 14).

Another important factor in increasing the efficiency of the photodynamic process is the greater accumulation of photosensitizers in the target tissue (15). To increase the accumulation of photosensitizers in tumor tissue, two approaches should be considered. These approaches include increasing blood half-life (blood circulation time) and targeting photosensitizers using the design of biocompatible nanocarriers (16, 17). 

Incorporating PSs into biocompatible nanocarriers can increase the half-life of PS in the body and control the release process of the photosensitizer in the living organism. Biocompatible nanostructures can be made in a wide range of sizes; however, particles with a diameter of 20–100 nm have better pharmacokinetic behavior and optimal intracellular uptake (18). Surface modification of nanostructures with hydrophilic polymers, such as polysaccharides, methoxy-polyethylene glycol (mPEG), polyoxamines, polylactic acids, and polyoxamers leads to increased biocompatibility, increased solubility in water, faster biodegradation, minimization of protein corona (adsorption of plasma proteins on the surface of nanostructures) and also prevents phagocytosis by macrophages and thus minimizes clearance by the reticuloendothelial system (19-21). Evaluation of the interaction between drugs and nanoparticles and plasma proteins is crucial to understanding the pharmacodynamics and pharmacokinetics of nanoparticle-ligand and its effect on the distribution of nanodrugs or drugs in target tissues. The biomolecular corona phenomenon is mainly caused by important proteins such as human serum albumin (HSA) and human holotransferrin (HTF) (22). 

The ideal nanocarriers should have special features such as long half-life, chemical stability, low toxicity, biodegradation capabilities, and high conjugation capacity. In particular, functionalized nanostructures with the ability to encapsulate or conjugate drugs are more advantageous than free drugs, providing benefits such as lower drug toxicity, higher targeting efficiency, and prolonged half-life, creating new paths to overcome challenges associated with chemotherapy and photodynamic therapy (23). One of the exciting candidates for photochemical internalization is gold nanostructures (AuNSs), which have received a lot of attention today due to their amazing properties. Their most important features include (i) Easy synthesis and unique optical properties: The biophysicochemical properties of AuNSs can be adjusted by changing the diameter (from 1 to 150 nm) and the shape (sphere, rod, nanoshell, nanostar, triangle, and nano-cage) as desired. The plasmon absorption peak of gold nanoparticles can be adjusted in a range of visible to infrared waves based on changes in the size and shape of the nanostructure. This feature has made them one of the most important agents in photodynamic and photothermal therapies (24). (ii) Chemical inert: AuNSs does not primarily cause acute cytotoxicity and has high biocompatibility (25). (iii) Surface modification: Amines and thiols could bind to the surface of AuNSs to provide a high cross-section for conjugation of AuNSs to some active groups such as biotin, proteins, nucleic acids, and peptides by the chemistry of gold-amine, thiol-gold, or electrostatic interactions (26).

The most important advantages of gold nanoparticles over other nanoparticles that led us to use this nanostructure in our study are the highly tunable optophysical properties, good adsorption cross-section for surface modification with multi-factor conjugation, simple and cheap synthesis, and low toxicity.

Although the use of nanoparticles as photosensitizing carriers can passively accumulate them in tumor tissue due to the effect of EPR, the internalization of these nanocarriers can be greatly improved by conjugation of a high-affinity targeting ligand (27). 

Folic acid (FA; also known as vitamin B9) is essential for cell proliferation and biosynthesis of nucleotide bases. FA (MW= 441 Da) is one of the most commonly employed targeting ligands with high overexpressed receptors on any type of cancer cells (such as breast, kidney, brain, ovary, colon, and lung). The main mechanisms of drug uptake via folate in non-malignant and malignant tissues are performed in three ways. (i) The main uptake pathway, which is distributed throughout the cell membrane and aids in the uptake of dietary folate, is through the reduced folate transporter (RFC). (ii) The second uptake pathway, which uses membrane proton gradients as intermediates for folate transport to cells, is through proton-coupled folate transporters (PCFTs). (iii) The last pathway is related to the transfer of folate through four folate glycopolypeptide receptors with molecular weights from 38 to 45 kDa. (FRα, FRβ, FRγ, and FRδ). All three proteins play a key role in folate uptake, however, only folate receptors (FRs) are the direct target of common anticancer drugs. Studies show that FRα is overexpressed in solid tumors such as lung, ovarian, and breast cancers. On the other hand, the distribution of FRα in noncancerous tissues in the body is limited to the expression of low levels in the apical surfaces of some organs such as the lung, kidney, and choroid plexus (28, 29).

The FA-conjugated drug nanocarrier is internalized through receptor-mediated endocytosis and delivers its drug content into the cell (30). 

Despite the benefits of targeting nanostructures by conjugation to folic acid, there are always challenges to using this nanocomplex. These challenges are due to the lack of a proper surface modification process before conjugation of folic acid to nanostructures.

If the primary surface modification process is ignored or not performed correctly (which includes coating the nanostructure with an optimized concentration of the appropriate coupling agent), it will reduce the loading efficiency of the secondary drug, cell uptake, and controllability of drug release.

In this study, for the first time, by surface modification of gold nanoparticles with 6-mercapto-1-hexanol, two agents of protoporphyrin IX and folic acid were conjugated simultaneously on the surface of nanoparticles and the targeted photochemical internalization was facilitated by this phenomenon. Finally, cell death due to the photochemical process of nanocomplex interaction on the HeLa cell line was investigated.

## Materials and Methods

***Chemicals***

Protoporphyrin IX (PPIX), sodium borohydride (NaBH_4_, 99.99% purity), hydrogen tetrachloroaurate (III) trihydrate (HAuCl_4_·3H_2_O, 99.5% purity), folic acid, N,N’-Dicyclohexylcarbodiimide (DCC), Medroxyprogesterone acetate (DMPA), MTT (3-[4,5-dimethylthiazol-2-yl]-2,5-diphenyltetrazolium bromide), Dimethyl sulfoxide (DMSO), trypan blue, streptomycin, penicillin, and trypsin–EDTA were purchased from the Sigma Aldrich Company. RPMI 1640 and fatal bovine serum (FBS) were provided by the Hyclone Laboratories (HyClone, Logan, UT, USA). Also, other chemical and biological reagents used in this project were purchased from Fluka (Switzerland) and Merck (Germany). 

***Instrumentations***

The main equipment used in this study were CO_2_ incubator, UV–Vis spectrophotometer (UNICO UV-2100), Transmission electron microscopic (TEM) system (Zeiss EM 900 model), and Elisa reader (AWARENESS, USA). In order to prepare the samples for TEM measurement, a drop of colloidal solution containing nanoparticles was placed on a carbon-coated copper grid. Then it was exposed to air for natural drying. By counting at least 300 particles under TEM, the size distribution of the final nanocomplex was estimated. Gold percentage in the gold nanoparticle samples was determined by the Shimadzu model AA-670 atomic absorption spectrometer. An incoherent light source (LUMACARE, 630 ± 25 nm) was used for the PDT procedure.

***Synthesis of 6-mercapto-1-hexanol conjugated with GNPs (MH-AuNP)***

For this purpose, sodium borohydride was used as a reducing agent for the synthesis of gold nanoparticles conjugated to mercapto-hexanol. First, a solution of HAuCl_4_·3H_2_O (13 mg, 0.03 mmol) in CH_3_OH (2 ml) was added to the stirring solution of 6-mercapto-1-hexanol (20 mg, 0.03 mmol) in CH_3_OH (1.5 ml). After stirring for 30 min, the freshly prepared solution of sodium borohydride (19 mg, 0.5 mmol) in CH_3_OH (1.5 ml) was added to the reaction mixture. As sodium borohydride was added, the reaction mixture turned dark brown, indicating the formation of gold nanoparticles. After two hours of mixing the solution, a centrifuge with a speed of 10000 rpm is used to prepare a solid precipitate of nanostructure and finally, it is washed with pure methanol (36). Thus, MH-AuNP nanoconjugates are obtained as a dark brown powder after drying. 

***Synthesis of PPIX/FA-MH conjugated with GNPs (PpIX/FA-MH-AuNP)***

After surface modification of gold nanoparticles, protoporphyrin IX (PpIX) and folic acid (FA) are bonded to the surface of the functionalized gold nanoparticles through the coupling reaction to produce the desired conjugated gold nanoparticles. In this process, MH-AuNP nanoconjugates are first mixed with N, N-dicyclohexylcarbodimide (DCC; 53 μmol, 11 mg) and dimethylaminopyridine (DMAP; 53 μmol, 6.5 mg) in tetrahydrofuran (THF; 1 ml). After a brief activation time (10 min), PpIX and folic acid in THF are added to the reaction mixture. The mixture was stirred at room temperature for 24 hr. After the solvent is removed under reduced pressure, the residue is washed with organic solvents, and finally, the gold nanoparticles conjugated to protoporphyrin IX and folic acid (FA / PpIX-MH-AuNP) are obtained (36).

***Optical studies of the prepared nanocompounds***

Ultraviolet-visible spectroscopy is one of the useful techniques for the optical characterization of nanomaterials. Other important applications of UV-Vis spectroscopy include quantitative or qualitative examination (confirmation) of the completion of the process of final nanostructured synthesis. To obtain the absorption spectrum and increase the solubility of the samples, the sample was first dissolved in a very small amount of DMSO and then reached the desired volume by deionized water. These data are evaluated based on quantitative changes in the absorption spectrum and displacement of the absorption peak of the sample. For this purpose, after preparation of sample solutions (free protoporphyrin and protoporphyrin/folic acid conjugated to gold nanoparticles), their absorption spectra were determined in the range of 200 to 800 nm by UNICO UV-2100 spectrophotometer (in a quartz cuvette with 1.0 cm path length). 

The use of Fourier transforms infrared (FTIR) spectroscopy has been considered to be one of the most effective techniques to study the surface chemical composition and to recognize the existence of different functional groups in nanomaterials. The mechanism of FTIR is based on the absorption of IR radiation by the irradiated material and then stimulation of molecules to transfer to a higher vibrational state. According to the wavelengths absorbed by the sample, its molecular structure can be understood. In this study, the FTIR technique was used to investigate the presence or absence of chemical bonds between Pp-IX and folic acid molecules with gold nanoparticles.

***Morphology and size studies of the prepared nanocompounds***

To study the morphology and size of the synthesized nanoparticles, Transmission electron microscopy (TEM) (Zeiss EM 900 model, accelerating voltage of 120 kV, line resolution of 0.3 nm) was used. To prepare the sample, first, a small drop of colloidal suspension (usually about 5 µl) is pipetted onto a carbon-coated copper grid and simply allowed to dry at room temperature. Then, using the recorded TEM image, the final size distribution of nanoparticles is determined by counting at least 300 particles. 

***Cell culture***

HeLa cells, which are derived from human cervical cancer, were purchased from the National Cell Bank of Iran (NCBI), Pasteur Institute of Iran. The cell line was cultured in 75 cm^3^ tissue culture flasks in RPMI 1640 containing fetal bovine serum (10%), streptomycin (100 μg/ml), and penicillin (100 units/ml) in a humidified incubator (Cellamate; Coda Filter-System). A carbon dioxide incubator with atmospheric conditions of 5% CO_2_ and temperature of 37 °C was used for cell culture. 

***PDT procedure***

Hela cells were seeded in 96-cell culture plates (2×10^4^ cells per cell). One day after cultivation, 200 μl of the main solution of PpIX/FA-MH-AuNP was added to the first and second plates containing Hela cells, respectively. Also, for the control test, 200 μl of gold nanoparticle solution and PpIX solution, separately is added to the third plate. The fourth plate is also used as the second control, without any treatment. In all cases, the concentrations of 2.5-20 μg/ml of PP-IX are tested. After 3 hr of incubation of the different treatment groups, the medium is removed and the cells are washed with sterile phosphate saline buffer (PBS). Afterward, the cells were exposed to different radiant exposure times (1, 5, and 10 min) using a LUMACARE light source. After irradiation of the target groups, in order to adjust the amount of FBS (based on 10%) in each well, a fresh culture medium (with 17% FBS) was added to the wells and placed in an incubator for 16 hr and then measured MTT was performed.

***Cell survival assay***

The viability of Hela cells was assessed 16 hr after each treatment. Before MTT assay, cells should be carefully examined under a microscope in order to check the process of cell division in the control and treatment groups and ensure the absence of any contamination. After a specified period of time, the previous cell culture medium was drained. After preparing the MTT solution (5 mg/ml), 50 microliters of MTT solution with a culture medium without FBS were added to the wells in dark conditions, then the plates were covered with aluminum foil and placed in the cell culture incubator. After 4 hr, the medium containing MTT was slowly drained and 200 μl of DMSO (dimethyl sulfoxide) solvent was added to each well to dissolve the formazan crystals. Then, to ensure the uniformity of the solutions, each plate was placed on a shaker for 5 min and their optical density was read with an ELISA reader. Also, in order to study the morphological changes of the cells, first Giemsa staining was done and it was examined using an optical microscope with imaging capability (Eclipse 3200; Nikon, Japan).

***Cell imaging***

To carry out the Giemsa staining process, first, the HeLa cells were grown to 80 % confluency in 25 cm^2^ flasks for 48 hr. Then, the HeLa cells were seeded at 10^5^ cells/well in 6-well tissue culture plates, and the plates were incubated overnight at 37 and 5 % CO_2_. Then, the cells were incubated with certain concentrations of PpIX and PpIX/FA-MH-AuNP (5 g/ml) for 4 hr. Afterward, according to the radiation condition, they were exposed for 10 min. After an additional 24 hr of incubation, the cells were gently washed once or twice with PBS and then fixed with cold methanol for 5 min. After a few minutes, the methanol was removed and the cells were air dried . The fixed cells in the wells were stained with fresh Giemsa staining solution for 6 min and then washed three times with sterile water (31). Finally, morphological changes were surveyed using a light microscope (eclipse 3200; Nikon, Japan).

***IC***_50_ ***and ED***_50_ ***calculation***

In order to compare the therapeutic effect of different groups (cytotoxicity of agents with or without radiation), two indices ED_50_ and IC_50_ were defined. The ED_50_ index was the amount of exposure required to cause 50% cell death, and the inhibitory concentration index of 50% index (IC_50_) means the amount of concentration required for 50% cell death. The IC_50_ and ED_50 _of the agents were calculated with the functions fitted and extrapolated on the data in the Masterplex software (four logistic parameters).

***Statistical analysis***

SPSS 16 software was used for data analysis. The survival curves of the treatment groups were plotted by Excel software. The normality of data was assessed by the nonparametric Kolmogorov-Smirnov test. Given the normality of data distribution, the Tukey test and one-way ANOVA were employed to compare mean differences. All data are presented as means ± SD. A value of *P*<0.05 was considered significant. Each experiment was repeated four times.

## Results

***Characterization of MH—AuNP and PPIX/FA-MH-AuNP***

**Figure 1:**
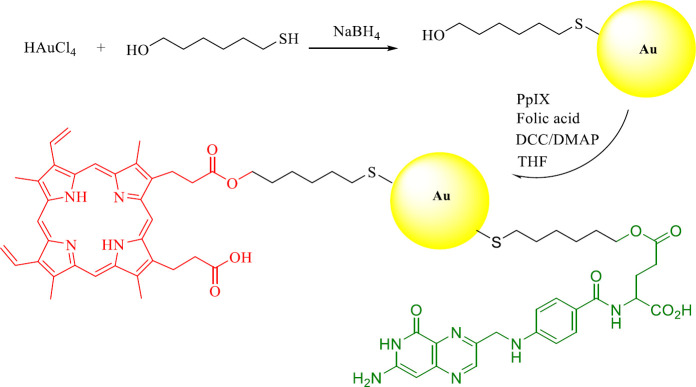
Synthesis of protoporphyrin and folic acid conjugated gold nanoparticles (PpIX/FA-MH-AuNP)

In this study, gold nanoparticles are first conjugated with protoporphyrin IX (PpIX) and then with folic acid (FA) for more effective targeting. These nanoparticles with an average diameter of about 7 nanometers are made by a coupling reaction. Also, protoporphyrin absorbs light at one of the absorption peaks (relative maximum or extremum; 630 nm), which causes photochemical reactions that eventually produce free radicals such as reactive oxygen species with high efficiency. This phenomenon shows that gold nanoparticles attached to protoporphyrin IX (PpIX-AuNPs) as well as folic acid-protoporphyrin IX (PpIX-AuNP-FA) can be used as plasmonic nanosensitizers to perform photochemical internalization in cancer research. To conjugate the PpIX and FA molecules to the gold surface, gold nanoparticles must be functionalized with 6-mercapto-1-hexanol. The photosensitizer and folic acid were attached to the surface of gold nanoparticles through a coupling reaction (Figure 1).

**Figure 2:**
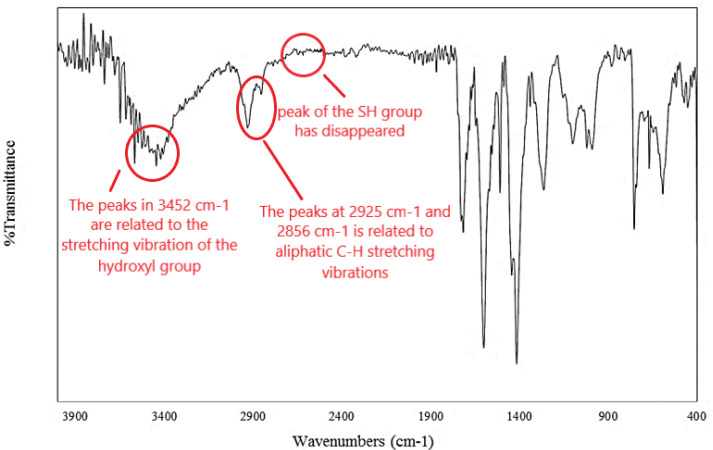
FT-IR spectrum of gold nanoparticles attached to 6- mercapto-1- hexanol

Using atomic absorption spectroscopy, the net amount of gold in the sample was 58%. Figure 2 shows the FT-IR spectrum of the dried sediments of the resulting gold nanoparticles. The peaks in 3452 cm^-1^ are related to the stretching vibration of the hydroxyl group. In addition, the presence of peaks at 2925 cm^-1^ and 2856 cm^-1^ is related to aliphatic C-H stretching vibrations. Also, the peak of the SH group, which appears in 2550-2560 cm^-1^, is not observed in the spectrum. Therefore, atomic absorption spectroscopy and FT-IR confirm the presence of 6-mercapto-1-hexanol on the surface of gold nanostructure.

**Figure 3:**
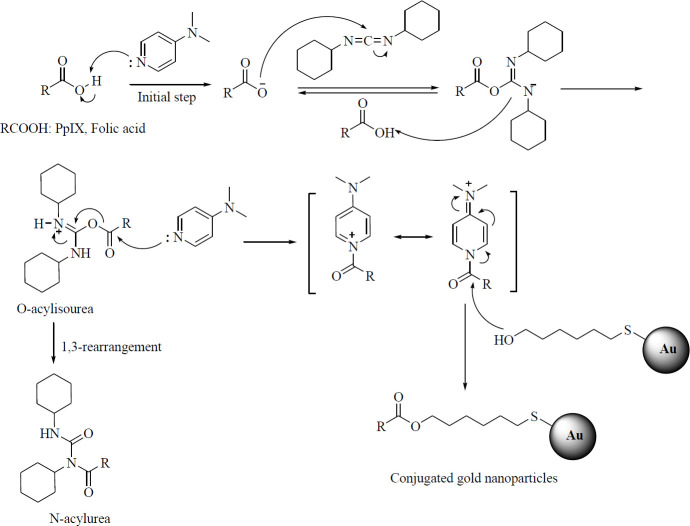
Mechanism of synthesis of conjugated gold nanoparticles in the presence of DCC and DMAP

DCC: Dicyclohexylcarbodiimide; DMAP: Dimethylaminopyridine

Figure 3 shows the surface modification of gold nanoparticles to prepare for the process of conjugation with photosensitizers. In this mechanism, N, N-dicyclohexyl carbo dimide (DCC) acts as a coupling agent and dimethylaminopyridine (DMAP) is involved in the steglich esterification process. The free carboxy groups of protoporphyrin and folic acid react with DCC in the presence of DMAP to obtain O-acylisourea. In this reaction, a small number of DMAP molecules act as initiators of the reaction by adsorbing proteins from the desired acid. In the next step, the free alcohol group attacks the activated carboxy groups on the surface of the gold nanoparticles. Because the reaction of esterification of carboxy groups with alcohols is usually slow, especially if the molecules are bulky, it is possible to carry out unwanted rearrangements of O-acylisourea and to produce the by-product of N-acylurea. As a result, the yield of the product is low and it becomes difficult to purify it naturally. To prevent this reaction from occurring, DMAP is used, which participates in the reaction mechanism as an acyl transport agent.

**Figure 4:**
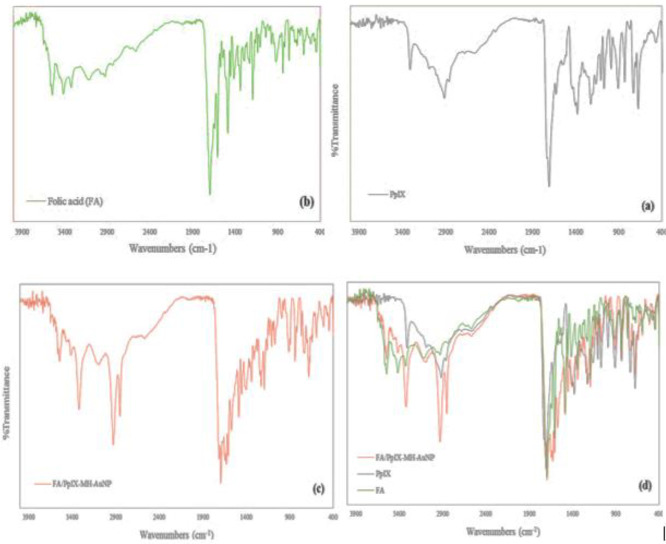
FT-IR spectra of samples a) free protoporphyrin, b) folic acid, c) gold nanoparticles attached to protoporphyrin and folic acid, and d) adaptation of three spectra (a), (b), and (c)

Using atomic absorption spectroscopy, the net amount of gold in the sample was reported to be 22%. Figure 4 shows the FT-IR spectrum of the dried deposits of the resulting gold nanoparticles in comparison with protoporphyrin and free folic acid. In the FT-IR spectrum of gold nanoparticles, the peaks located in 3550 cm^-1^, 3412 cm^-1^, and 3329 cm^-1^ correspond to the stretching vibrations of the first and second groups of NH amine groups of folic acid and protoporphyrin conjugated on gold nanoparticles and peaks located in 1712 cm^-1^, 1707 cm^-1^, 1693 cm^-1^, and 1683 cm^-1^ belong to the stretching vibrations of their acidic and esterified carbonyl groups. In addition, the presence of broad peaks in 3101 cm^-1^ and 2565 cm^-1^ are related to the stretching vibrations of acidic and alcoholic OH groups. Also, the presence of peaks in 2928 cm^-1^ and 2848 cm^-1^ are related to the stretching vibrations of C-H aliphatic 6-mercapto-1-hexanol. Therefore, atomic absorption spectroscopy and FT-IR confirm the presence of protoporphyrin and folic acid on the surface of gold nanoparticles.

The amount of protoporphyrin and folic acid loaded on the gold surface is calculated by absorption spectroscopy. By this technique, the loading ratio of the folic acid molecule to protoporphyrin on the surface of gold nanoparticles is 1.3.

**Figure 5:**
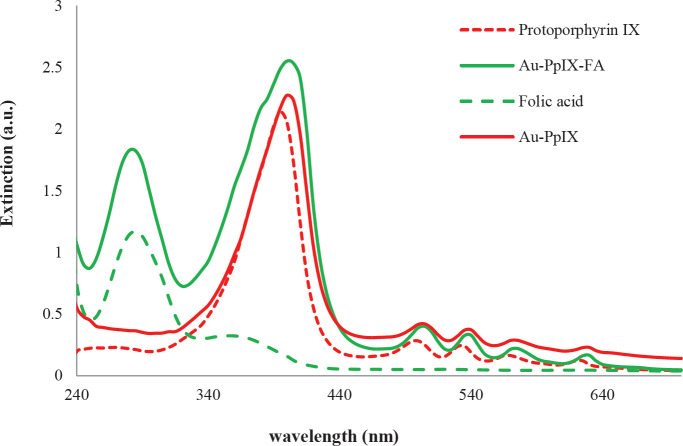
Absorption spectra of free folic acid, PpIX, Au-PpIX, and Au-PpIX/FA-MH. (to obtain the absorption spectrum and increase the solubility of the samples, the sample is first dissolved in a very small amount of DMSO and then reached the desired volume by deionized water)

The preferred photosensitizers in *in vivo* applications of photodynamic therapy are those that effectively absorb red light (> 600 nm). Such wavelengths have the power to penetrate deeply into most human tissues. In addition, red/infrared light is partially absorbed by the internal components of body tissues, thereby minimizing the risk of general complications of photodynamic therapy. The absorption spectra of PpIX/FA-MH-AuNPs are shown in Figure 5. The relative absorption peak (extremum) of PpIX after conjugation with gold nanoparticles is 630 nm, which is 10 nanometers shifted towards the red region compared with the relative absorption peak (extremum) of PpIX before the conjugation process. Also, the intensity of protoporphyrin Q peaks has changed. These phenomena may be due to changes in the environment. The displacement of the adsorption peak towards the red region is due to the individual surface plasma coupling of the nanoparticles in the aggregated structures. 

Also, some different studies show that low displacements (about 10 nm) along with changes in absorption coefficient are related to changes in the spatial conformation of conjugated molecules to the surface of nanoparticles and changes in spin states (32).

**Figure 6:**
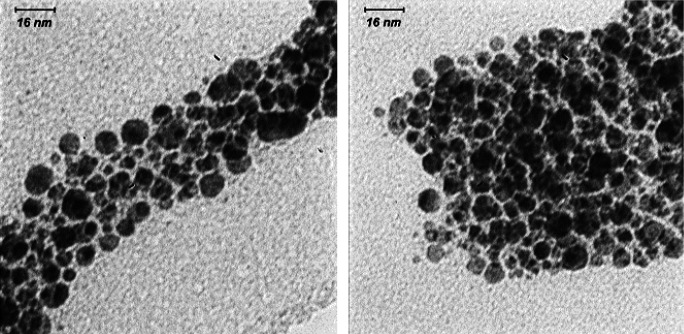
TEM images of gold nanoparticles attached to folic acid-protoporphyrine

Figure 6 shows TEM images of PpIX/FA-MH-AuNP. The TEM image is a sample of gold nanoparticles attached to folic acid-protoporphyrin IX (PpIX-GNP-FA). The size distribution of the final PpIX/FA-MH-AuNP nanostructure in this process can usually be determined by counting about 300 particles. These spherical nanostructures have an average diameter of 7 nm.

***Photochemical internalization on cancer cell–in vitro***

**Figure 7:**
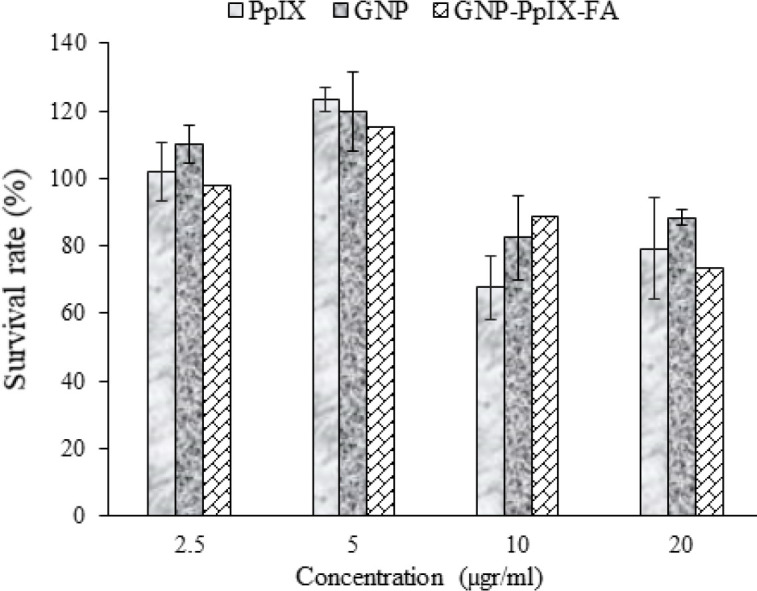
Cell survival before PDT. The effect of dark toxicity of each component, separately, (2.5–5 μg/ml of protoporphyrin) (number of repetitions; n=4)

In order to investigate the efficiency of photochemical internalization of PpIX/FA-MH-AuNP in photodynamic therapy, various parameters such as concentration and irradiation time were investigated. The results of the cytotoxicity test (non-irradiated groups) are shown in Figure 7. The results show that increasing the concentration of PpIX from 5 to 20 μg/ml leads to significant inhibition of cell proliferation potential (*P*<0.05). A similar pattern is also observed in the presence of similar concentrations of gold nanoparticles. While PpIX/FA-MH-AuNPs at all concentrations reduced cell proliferation compared with the control group (*P*<0.05). However, a significant increase in cell proliferation was observed at a concentration of 5 μg/ml of PpIX/FA-MH-AuNPs. Due to the small size of gold nanoparticles, it is predictable that in Hela cells, through endocytosis processes, conjugated nanoparticles with photosensitizer enter the cell from outside the cell.

So far, valuable studies have been conducted on the biochemical and pharmacodynamic behavior of protoporphyrin IX on cancer cell division. The results show that PpIX concentration and cell density (cells/ml) are important parameters in determining the inhibitory or proliferative behavior of cancer cells. 

**Figure 8:**
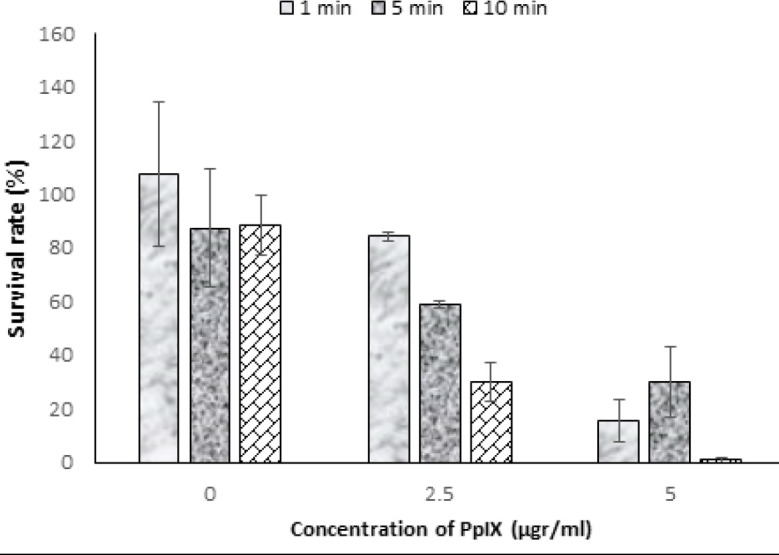
Cell survival after PDT (2.5–5 μg/ml of PpIX). (number of repetitions; n=4)

As shown in Figure 8, in the absence of photosensitizer, no significant changes were observed with increasing irradiation time in the initial minutes. An equally significant decrease in cell viability was reported after 5 and 10 min of exposure (*P*<0.041). But in the presence of PpIX, cell death is significant by increasing drug concentration in each light dose or increasing the light dose in a specific drug concentration (*P*<0.034).

**Figure 9:**
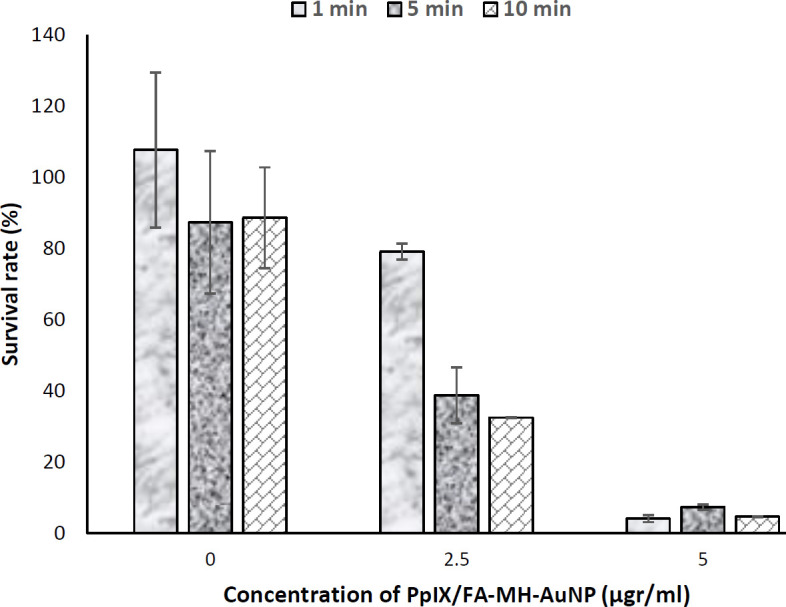
Cell survival rate after photochemical internalization in the presence of PpIX/FA-MH-AuNP (2.5 and 5 μg/ml) on HeLa cells after exposure for 1, 5, and 10 min (number of repetitions; n=4)

In general, performing photochemical internalization in the presence of PpIX/FA-MH-AuNPs, as shown in Figure 9, reduces cell survival. However, by increasing the irradiation time up to 10 min at a concentration of 5 μg/ml, no significant change in cell survival was observed.

**Figure 10:**
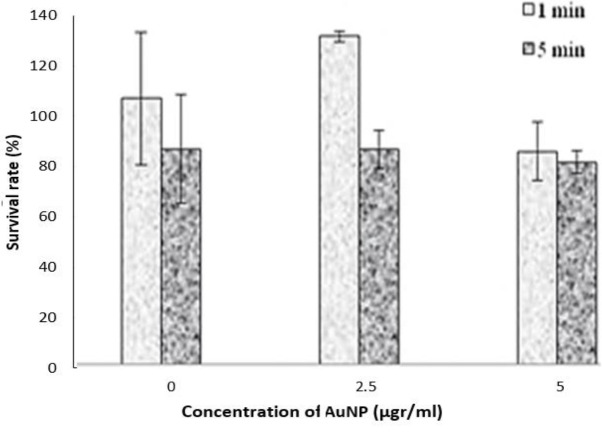
Cell survival rate after photochemical internalization in the presence of gold nanoparticles (2.5 and 5 μg/ml) on HeLa cells after exposure for 1 and 5 min (number of repetitions; n=4)

Figure 10 shows that after applying 1 min of irradiation (wavelength 630 nm) at a concentration of 5 μg/ml, a significant decrease in cell survival is observed (*P*<0.001, about 90%). Under these conditions, with increasing irradiation time, no significant decrease in cell survival is observed. The ED_50_ (dose required to induce 50% cell death) for PpIX and PpIX/FA-MH-AuNPs at 2.5 μg/ml is 6.7 and 5.34 min, respectively. 

**Figure 11:**
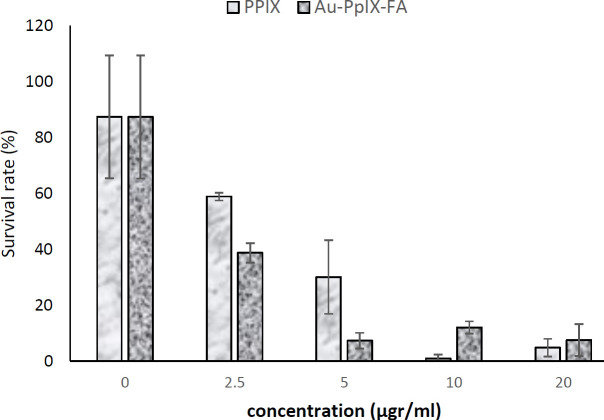
Cell survival rate after photochemical internalization in the presence of PPIX and PPIX/FA-gold nanoparticles (2.5 –20 μg/ml) on HeLa cells after exposure for 5 min (number of repetitions; n=4)

The effect of 5 min of exposure to PpIX and PpIX/FA-MH-AuNP is shown in Figure 11. The results show that increasing the concentration of PpIX and PpIX/FA-MH-AuNP up to 10 μg/ml in the presence of 5 min of irradiation significantly reduces cell survival. However, increasing the concentration of these agents from 10 to 20 μg/ml does not affect cell survival. Because increasing the concentration of the drug reduces cell survival to zero. The required concentrations of photosensitizer to induce 50% cell death (IC_50_) for PpIX and PpIX/FA-MH-AuNPs are predicted to be 3.7 and 2.15 μg/ml, respectively. according to the results of previous studies, drug concentrations and cell densities are key parameters in cell proliferation or death. 

Increasing the PpIX concentration to 20 μg/ml causes significant inhibition of cell proliferation. In the case of PpIX/FA-MH-AuNPs, an increase in cell proliferation at a concentration of 10 μg/ml of conjugated gold nanoparticles is observed, which may be due to the different mechanism of nanoparticles’ entry from the extracellular environment into the cell, at different concentrations of conjugated nanoparticles. In other words, the structural properties of nanoparticles such as size, type of coating, and how the drug is loaded on the surface of nanoparticles can determine the optimal concentration *in vitro*. Accordingly, in the case of conjugated nanoparticles synthesized in this study, according to the results, the concentration of 5 μg/ml of conjugated nanoparticles is the optimal concentration for inhibiting cell proliferation. In this regard, in the continuation of research and treatment, concentrations of 5 and 2.5 μg/ml were selected and evaluated. 

**Figure 12:**
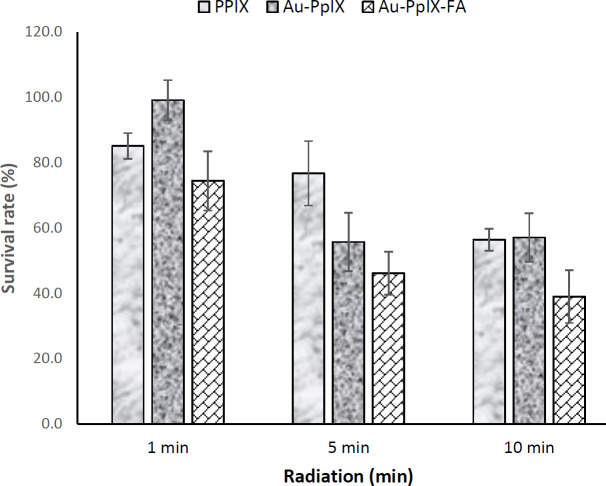
Cell survival from photochemical internalization after 30 min of cell incubation with the drug. PpIX and PpIX/FA-MH-AuNP (2.5 μg/ml) on Hela cell survival after exposure for 1, 5, and 10 min. (data are measured relative to the control group) (number of repetitions; n=4)

Also, to investigate the effect of folic acid on photochemical internalization and to make a significant difference between the data obtained from treatment with PpIX and PpIX/FA-MH-AuNPs, the incubation time of the drug was reduced from 3 hr to 30 min. As can be seen in Figure 12, the results indicate that at each light dose, cell death in the presence of PpIX/FA-MH-AuNPs was significantly reduced compared with unconjugated conditions (*P*<0.001). Under these conditions, ED_50_ for PpIX and PpIX-MH-AuNP and PpIX/FA-MH-AuNP at the concentration of 2.5 μg/ml is 8.9, 9.1, and 6.17 min, respectively.

***Investigation of microscopic changes in cell morphology in the process of photochemical internalization***

Apoptosis is the main form of cell death caused by radiation treatments and photodynamic therapy (33). In clinical processes, apoptotic death of cancer cells is preferred to their necrotic death because it causes fewer adverse immunological reactions in tissues. The probability of the apoptosis process depends on various parameters such as the type of cell line, the chemical formula of photosensitizer, and irradiation conditions (34). Studies on the ratio of apoptosis to necrosis show that lower optical doses and concentrations of PpIX are preferred due to the increased probability of cellular apoptosis. In 2009, Cohen *et al*. investigated the performance of silica nanoparticles encapsulated with PpIX in HeLa cells (35). The results showed that PDT can simultaneously cause apoptosis and necrosis of Hela cancer cells.

DMSO: Dimethyl sulfoxide

PPIX: Protoporphyrin IX

**Figure 13:**
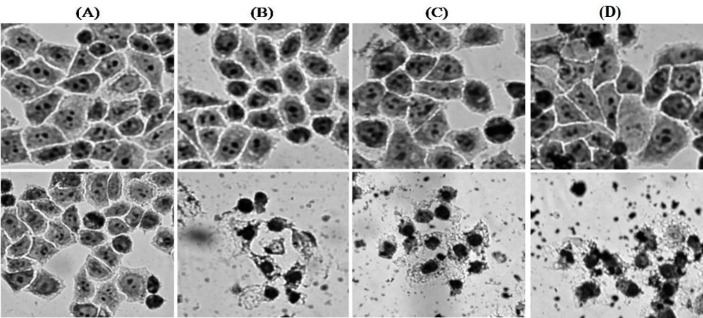
Microscopic images of cell morphology by Giemsa staining with 400 × magnification before exposure (top row) and after 10 min of exposure with an output wavelength of 630 nm (bottom row). (A) Hela cells, (B) Hela cells containing 5 μg/ml PpIX, (C) 5 μg/ml PpIX-conjugated nanoparticles, and (D) 5 μg/ml PpIX/FA-MH-AuNP

## Discussion

The presence of properties such as relative stability of the gold-thiol bond in an aqueous medium and its reduced stability inside the cell (due to the concentration of glutathione inside the cell) have made gold nanoparticles a suitable option for drug delivery to the cell. In the present study, 6-mercapto-1-hexanol was selected as a more effective binder after considering the cell line type and conjugated gold nanostructure concentration. This selection is based on the pervious study (36). On the reactions of gold nanoparticles coupled with different compounds as well as the results of *in vitro* experiments in the presence of conjugated gold nanoparticles with different binders, to selectively target cancer cells. Then, after much trial and error, PpIX/FA-MH-AuNPs were optimized using 6-mercapto-1-hexanol linker and synthesized to apply photochemical internalization methods to cervical cancer cells.

The cellular uptake rate of gold nanoparticles depends on their surface modification, Zeta potential, size, and hydrophobicity/hydrophilicity. In addition, tumor cells require protoporphyrin in order to regulate the cell division cycle and DNA nucleotide synthesis. Also, the surface charge sign on gold nanoparticles is very effective on the affinity index between the particles and the cell membrane. Therefore, due to the interaction of the surface charge and also the interaction of the functional groups with the cellular receptor, the uptake of PpIX/FA-MH-AuNPs into the cell is done effectively.

On the other hand, folic acid is a ligand that can bind to bioactive agents, especially the folate receptor (FR) of cancer cells. Folate receptors exist in three main forms: FR-α, FR-β, and FR-γ. The FR-α form is expressed by many tumor cells such as ovarian, endometrial, colorectal, breast, lung, kidney, and brain metastases. In addition, due to its high folic acid stability, compatibility with aqueous and organic solvents, low cost, non-toxic properties, ability to conjugate with many molecules, and low molecular weight, it has attracted widespread attention in targeted cancer therapies. Therefore, the proper synthesis for the binding of folic acid to molecules is of great interest due to the development of targeted delivery systems (39-41).

Protoporphyrin IX is naturally the main component of all types of cytochromes, hemoglobin, and some known biological molecules that are essential for cell life. PpIX, on the other hand, PpIX is a second-generation photo/radio sensitizer, which can be produced in the body using 5-aminoluronic acid as a precursor and through a biosynthetic pathway (HEME). This agent strongly emits a fluorescence signal and rapidly undergoes photochemical degradation under the production of free radicals (such as singlet oxygen). Although PpIX is used as a PS in PDT, its direct use is limited due to its low solubility in biological media. To date, various nanocarriers for photosensitizers have been synthesized in order to increase water solubility and facilitate drug delivery (42). Among the common carriers, gold nanostructures have attracted much attention in theranostic applications due to their unique optophysical properties.

By combining the results of cell survival of different treatment groups, it can be concluded that PpIX/FA-MH-AuNP are more efficient in uptake to the cell due to the presence of a folic acid ligand. Thus, folic acid on gold nanoparticles has been able to act as a targeting agent in the photochemical internalization of Hela cells.

In the present study, the morphological deviations of treated cells caused by photochemical internalization were investigated by Giemsa staining. Figure 13 shows the results of changes in cell morphology using a light microscope. These microscopic images were recorded by a digital camera. The images show that cell death has occurred in a significant number of cells. The nucleus of most cells is divided into several distinct bold components. Also, the color intensity in the nucleus is stronger than in living cells, which indicates the occurrence of an apoptotic event. Images of PpIX-conjugated nanoparticles have been reported in our previous work (36).

## Conclusion

Photochemical internalization is one of the new branches of light therapy that combines chemotherapy and targeted photodynamic therapy (TPDT). One of the key factors used in targeted photochemical internalization is the use of ligand-functionalized nanoparticles that can target specific proteins or receptors for cancer cells. In this study, synthesized gold nanoparticles were first functionalized with 6-mercapto-1-hexanol to increase the drug loading efficiency and also to improve the targeted endocytosis process of the nanostructure. Then the protoporphyrin IX and folic acid were conjugated to the functionalized gold nanoparticle surface and a photochemical internalization process was performed on this nanocomplex in the Hela cell line. The results show that the design of this nanostructure and its application in the photochemical process of internalization has led to an increase in specific light toxicity on cancer cells compared with the conventional process of photodynamic therapy.

Research perspectives on photochemical internalization can be based on the use of multi-physics simulation software (such as COMSOL), molecular dynamics modeling, or docking to optimize and accurately interpret existing events. The use of molecular docking software can better identify the strengths and weaknesses of molecular mechanisms and be very effective in achieving ideal interactions at the cellular and DNA levels. Also, the use of multispectroscopic methods (such as viscosity measurements, circular dichroism spectroscopy, fluorescence competition displacement assay, resonance light scattering spectroscopy, thermal denaturation evaluation, etc.) to investigate the physical and optical behavior of the interaction can be effective in optimizing the optophysical parameters in the treatment (37, 38).

## Authors’ Contributions

NA Designed the experiments; NA and AI Performed experiments, collected data, and discussed the results and strategy; NA, HE, and AS Supervised, directed, and managed the study; NA, AI, HE, and AS Approved the final version to be published.

## Conflicts of Interest

There are no conflicts of interest to report.

## References

[1] Agostinis P, Berg K, Cengel KA, Foster TH, Girotti AW, Gollnick SO (2011). Photodynamic therapy of cancer: an update. CA Cancer J Clin.

[2] Chen Y, Li W, Zhou J, Wen Y, Miao X, Xiong L (2014). [Molecular mechanism of photodynamic therapy]. J Cent South Univ (Med Sci).

[3] Castano AP, Demidova TN, Hamblin MR (2004). Mechanisms in photodynamic therapy: part one-photosensitizers, photochemistry and cellular localization. Photodiagnosis Photodyn Ther.

[4] Kruger CA, Abrahamse H (2018). Utilisation of targeted nanoparticle photosensitiser drug delivery systems for the enhancement of photodynamic therapy. Molecules.

[5] Zhang X, Chen X, Guo Y, Jia HR, Jiang YW, Wu FG (2020). Endosome/lysosome-detained supramolecular nanogels as an efflux retarder and autophagy inhibitor for repeated photodynamic therapy of multidrug-resistant cancer. Nanoscale Horiz.

[6] Shete HK, Prabhu RH, Patravale VB (2014). Endosomal escape: a bottleneck in intracellular delivery. J Nanosci Nanotechnol.

[7] Petros RA, DeSimone JM (2010). Strategies in the design of nanoparticles for therapeutic applications. Nat Rev Drug Discov.

[8] Bareford LM, Swaan PW (2007). Endocytic mechanisms for targeted drug delivery. Adv Drug Deliv Rev.

[9] Yaghini E, Dondi R, Tewari KM, Loizidou M, Eggleston IM, MacRobert AJ (2017). Endolysosomal targeting of a clinical chlorin photosensitiser for light-triggered delivery of nano-sized medicines. Sci Rep.

[10] Luo Z, Cai K, Hu Y, Li J, Ding X, Zhang B (2012). Redox‐responsive molecular nanoreservoirs for controlled intracellular anticancer drug delivery based on magnetic nanoparticles. Adv Mater.

[11] Omata D, Negishi Y, Hagiwara S, Yamamura S, Endo-Takahashi Y, Suzuki R (2011). Bubble liposomes and ultrasound promoted endosomal escape of TAT-PEG liposomes as gene delivery carriers. Mol Pharm.

[12] Ohtsuki T, Miki S, Kobayashi S, Haraguchi T, Nakata E, Hirakawa K (2015). The molecular mechanism of photochemical internalization of cell penetrating peptide-cargo-photosensitizer conjugates. Sci Rep.

[13] Jerjes W, Theodossiou TA, Hirschberg H, Høgset A, Weyergang A, Selbo PK (2020). Photochemical internalization for intracellular drug delivery From basic mechanisms to clinical research. J Clin Med.

[14] Selbo PK, Bostad M, Olsen CE, Edwards VT, Høgset A, Weyergang A (2015). Photochemical internalisation, a minimally invasive strategy for light-controlled endosomal escape of cancer stem cell-targeting therapeutics. Photochem Photobiol Sci.

[15] Supplitt S, Knap B, Przystupski D, Saczko J, Kędzierska E, Knap-Czop K (2018). Photodynamic therapy – mechanisms, photosensitizers and combinations. Biomed Pharmacother.

[16] Yoo JW, Chambers E, Mitragotri S (2010). Factors that control the circulation time of nanoparticles in blood: challenges, solutions and future prospects. Curr Pharm Des.

[17] Sztandera K, Gorzkiewicz M, Klajnert-Maculewicz B (2019). Nanocarriers in photodynamic therapy—in vitro and in vivo studies. Wiley Interdiscip Rev Nanomed Nanobiotechnol.

[18] Lombardo D, Kiselev MA, Caccamo MT (2019). Smart nanoparticles for drug delivery application: development of versatile nanocarrier platforms in biotechnology and nanomedicine. J Nanomater.

[19] Natarajan S, Harini K, Gajula GP, Sarmento B, Neves-Petersen MT, Thiagarajan V (2019). Multifunctional magnetic iron oxide nanoparticles: diverse synthetic approaches, surface modifications, cytotoxicity towards biomedical and industrial applications. BMC Mater.

[20] Chen D, Ganesh S, Wang W, Amiji M (2020). Protein corona-enabled systemic delivery and targeting of nanoparticles. AAPS J.

[21] Qie Y, Yuan H, Von Roemeling CA, Chen Y, Liu X, Shih KD (2016). Surface modification of nanoparticles enables selective evasion of phagocytic clearance by distinct macrophage phenotypes. Sci Rep.

[22] Sharifi-Rad A, Mehrzad J, Darroudi M, Saberi MR, Chamani J (2021). Oil-in-water nanoemulsions comprising berberine in olive oil: biological activities, binding mechanisms to human serum albumin or holo-transferrin and QMMD simulations. J Biomol Struct Dyn.

[23] Hong EJ, Choi DG, Shim MS (2016). Targeted and effective photodynamic therapy for cancer using functionalized nanomaterials. Acta pharmaceutica Sinica B.

[24] Kim HS, Lee DY (2018). Near-infrared-responsive cancer photothermal and photodynamic therapy using gold nanoparticles. Polymers.

[25] Kumar V, Ganesan S (2013). Size-dependent in vitro cytotoxicity assay of gold nanoparticles. Toxicol Environ Chem.

[26] Chen Y, Xianyu Y, Jiang X (2017). Surface modification of gold nanoparticles with small molecules for biochemical analysis. Acc Chem Res.

[27] Li L, Huh KM (2014). Polymeric nanocarrier systems for photodynamic therapy. Biomater Res.

[28] Matherly LH, Goldman DI (2003). Membrane transport of folates. Vitam Horm.

[29] Zhao R, Diop-Bove N, Visentin M, Goldman ID (2011). Mechanisms of membrane transport of folates into cells and across epithelia. Annu Rev Nutr.

[30] Zwicke GL, Mansoori GA, Jeffery CJ (2012). Utilizing the folate receptor for active targeting of cancer nanotherapeutics. Nano Rev.

[31] Sakalar C, Yuruk M, Kaya T, Aytekin M, Kuk S, Canatan H (2013). Pronounced transcriptional regulation of apoptotic and TNF-NF-kappa-B signaling genes during the course of thymoquinone mediated apoptosis in HeLa cells. Mol Cell Biochem.

[32] Chamani J, Moosavi-Movahedi AA (2006). Effect of n-alkyl trimethylammonium bromides on folding and stability of alkaline and acid-denatured cytochrome c: a spectroscopic approach. J Colloid Interface Sci.

[33] Agarwal ML, Clay ME, Harvey EJ, Evans HH, Antunez AR, Oleinick NL (1991). Photodynamic therapy induces rapid cell death by apoptosis in L5178Y mouse lymphoma cells. Cancer Res.

[34] Dahle J, Mikalsen SO, Rivedal E, Steen HB (2000). Gap junctional intercellular communication is not a major mediator in the bystander effect in photodynamic treatment of MDCK II cells. Radiat Res.

[35] Qian J, Gharibi A, He S (2009). Colloidal mesoporous silica nanoparticles with protoporphyrin IX encapsulated for photodynamic therapy. J Biomed Opt.

[36] Eshghi H, Sazgarnia A, Rahimizadeh M, Attaran N, Bakavoli M, Soudmand S (2013). Protoporphyrin IX-gold nanoparticle conjugates as an efficient photosensitizer in cervical cancer therapy. Photodiagnosis Photodyn Ther.

[37] Askari A, Mokaberi P, Dareini M, Medalian M, Pejhan M, Erfani M (2021). Impact of linker histone in the formation of ambochlorin-calf thymus DNA complex: multi-spectroscopic, stopped-flow, and molecular modeling approaches. Iran J Basic Med Sci.

[38] Sohrabi T, Asadzadeh-Lotfabad M, Shafie Z, Tehranizadeh ZA, Saberi M-R, Chamani J (2021). Description of the calf thymus DNA-malathion complex behavior by multi-spectroscopic and molecular modeling techniques: EMF at low and high frequency approaches. Iran J Basic Med Sci.

[39] Kelemen LE (2006). The role of folate receptor α in cancer development, progression and treatment: cause, consequence or innocent bystander?. Int J Cancer.

[40] Holmes RS, Zheng Y, Baron JA, Li L, McKeown-Eyssen G, Newcomb PA (2010). Use of folic acid–containing supplements after a diagnosis of colorectal cancer in the colon cancer family registry. Cancer Epidemiol Biomarkers Prev.

[41] Yan S, Huang Q, Chen J, Song X, Chen Z, Huang M (2019). Tumor-targeting photodynamic therapy based on folate-modified polydopamine nanoparticles. Int J Nanomedicine.

[42] Sachar M, Anderson KE, Ma X (2016). Protoporphyrin IX: the good, the bad, and the ugly. J Pharmacol Exp Ther.

